# MIKC-Type MADS-Box Gene Analysis Reveals the Role of *PlSOC1* in Bud Dormancy Transition in Herbaceous Peony

**DOI:** 10.3390/plants14060928

**Published:** 2025-03-15

**Authors:** Qiaoyu Huang, Xiaoxuan Chen, Shuyun Zhong, Shuangzhe Wu, Junhong Guo, Qiyao Wang, Jiahe Li, Danqing Li, Yiping Xia, Jiaping Zhang, Xiaobin Wang

**Affiliations:** 1 Jiangxi Provincial Key Laboratory for Postharvest Storage and Preservation of Fruits & Vegetables, College of Agronomy, Jiangxi Agricultural University, Nanchang 330045, China; qiaoyu_huang@163.com; 2 College of Agriculture and Biotechnology, Zhejiang University, Hangzhou 310058, Chinaypxia@zju.edu.cn (Y.X.); 3 Department of Landscape Architecture, Zhejiang Sci-Tech University, Hangzhou 310018, China; danqingli@zju.edu.cn

**Keywords:** herbaceous peony, MIKC-type genes, bud dormancy transition, *SOC1*

## Abstract

The MIKC-type MADS-box (MIKC) gene family is essential for controlling various plant developmental processes, including flowering time and dormancy transitions. Although the MIKC gene family has been widely studied across different plants, its characterization and functional study in herbaceous peony remain limited. In this study, 19 *Paeonia lactiflora* Pall. MIKC-type (PlMIKC) genes were identified from the transcriptome of a low-chilling requirement *Paeonia lactiflora* Pall. cultivar ‘Hang Baishao’. These MIKC genes were categorized into seven clades: six were classified as MIKC^C^-type, including FUL/AP1, DAM, PI, AGL18, AGL12, AG, and SOC1, and one, AGL30, was classified as MIKC*-type. Notably, the FLC clade genes were absent in *Paeonia lactiflora* Pall. The PlMIKC genes were predominantly localized to the nucleus, and their sequences contained highly conserved MADS and K-domains. Phylogenetic analysis demonstrated that PlMIKC genes share a strong evolutionary affinity with the MIKC genes from grapevine (*Vitis vinifera*) and poplar (*Populus trichocarpa*). A low-temperature-induced bud dormancy transition (BDT) experiment revealed that PlMIKC genes, such as *PlFUL* and *PlDAM*, were highly expressed during dormancy maintenance, while *PlSOC1*, *PlAGL12*, and *PlAGL30* were upregulated during BDT. Additionally, the transient overexpression of *PlSOC1* in ‘Hang Baishao’ significantly accelerated BDT and promoted bud break, suggesting that *SOC1*, traditionally linked to flowering regulation, also plays a key role in dormancy transition. Since limited literature on the MIKC gene family is currently available in herbaceous peony, this study expands the knowledge of the MIKC genes in *Paeonia lactiflora* Pall. and offers valuable insights into the molecular regulation of bud dormancy in response to low temperatures.

## 1. Introduction

Perennial plants in boreal and temperate regions have developed adaptive growth strategies that enable them to cycle annually between growth and dormancy, protecting shoot apical meristems from adverse environmental conditions [1]. A key trait of perennials is their capacity to remain dormant, during which the meristem temporarily becomes unresponsive to growth-promoting signals until dormancy is lifted [2]. Throughout the life cycle of perennial plants, buds progress through distinct dormancy stages: paradormancy, endodormancy, and ecodormancy [3]. Notably, the transition from endodormancy to ecodormancy is crucial for subsequent bud break and flowering under favorable spring conditions [4]. Understanding the mechanisms governing bud dormancy transition (BDT) is essential for addressing significant agricultural challenges, particularly issues like failed bud break and blooming of perennials growing in the subtropical zone [5,6,7,8].

MADS-box transcription factors are distinguished by the MADS domain, a highly conserved region located at the N-terminal of the protein [9]. The MADS-box gene family, comprising type I and type II (MIKC-type) genes, is crucial for plant growth and development, with MIKC-type genes serving as key regulators of bud dormancy and flowering [10,11]. MIKC-type genes are further categorized into two subgroups, MIKC^C^ and MIKC*, based on sequence variations in the intervening region [12]. In *Arabidopsis thaliana*, MIKC^C^ genes are grouped into 12 phylogenetic clades, including AP1/FUL, SOC1, AG, SVP, AGL15 (AGL18), AP3/PI, SEP, AGL6, FLC, BS, AGL12, and AGL17 [13,14]. The analysis of increasing transcriptome data indicates that MIKC genes are modulated by environmental signals and play a pivotal role in regulating bud dormancy [15,16]. Among these MIKC genes, the *Dormancy-associated MADS-box* (*DAM*) gene, a homolog of the *Arabidopsis SHORT VEGETATIVE PHASE* (*SVP*) gene, was first identified in a non-dormant evergrowing peach (*Prunus persica*) mutant [17] and later recognized as a master regulator of dormancy induction and maintenance in perennial species, such as peach [18], leafy spurge (*Euphorbia esula*) [19], pear (*Pyrus pyrifolia*) [20], and apricot (*Prunus mume*) [21].

In contrast to the *DAM* gene, another MIKC homolog, *Arabidopsis SUPPRESSOR OF OVEREXPRESSION OF CO1* (*SOC1*), has been shown to facilitate bud dormancy release and bud break in tree peony (*Paeonia suffruticosa*) [22] and kiwifruit (*Actinidia chinensis*) [23]. In hybrid aspen (*Populus tremula* x *Populus tremuloides*), the *APETALA1* (*AP1*) gene regulates seasonal growth cessation via the *CONSTANS* (*CO*)/*FLOWERING LOCUS T* (*FT*) module [24]. Additionally, a newly identified MIKC gene, the *low-temperature-induced MADS-box* (*LIM1*) gene, has been shown to activate the gibberellic acid (GA) pathway, facilitating dormancy release by reducing callose accumulation and reopening plasmodesmata [25]. MIKC genes are also involved in responding to various stresses. In *Arabidopsis*, *AGL16* negatively regulates drought resistance by affecting stomatal density and movement [26]. *AGL21* is responsive to various environmental stresses, with its overexpression resulting in increased sensitivity to abscisic acid, salt, and osmotic stresses [27]. Moreover, MIKC genes are also crucial for regulating flower development and flowering time [14]. In tomato (*Solanum lycopersicum*), the overexpression of the MIKC gene *Tomato MADS 8* (*TM8*) led to abnormal stamens and poorly viable pollen [28]. In *Arabidopsis*, key MIKC genes such as *SOC1* [29], *FLOWERING LOCUS C* (*FLC*) [30], *AGAMOUS-LIKE 18* (*AGL18*) [31], *AGL24* [32], and *SVP* [33] have been identified as crucial regulators of flowering time. Nevertheless, the mechanisms by which MIKC genes regulate bud dormancy in herbaceous perennials remain poorly understood.

Herbaceous peony (*Paeonia lactiflora* Pall.), belonging to the Paeoniaceae family, is a versatile ornamental and oil crop cultivated worldwide, known for its vibrant flowers and promising oilseed production [34,35]. The overwintering buds of herbaceous peony are generated from underground parts in autumn and enter endodormancy under a short daylength and low temperatures [36]. Sufficient low temperatures are needed in winter to complete the transition from endodormancy to ecodormancy [8]. However, global warming has resulted in increasingly frequent warm winters, reducing the accumulation of chilling temperatures necessary for the dormancy transition of herbaceous peony [36,37]. This disruption hampers subsequent growth and development, ultimately leading to reduced crop yields, particularly in southern China [37,38]. Current research has predominantly focused on understanding the processes of bud dormancy in woody plants, while the mechanisms governing these processes in herbaceous peony remain largely unexplored [39]. Although our previous studies on physiological responses and gene expression have identified several MIKC gene homologs, such as *SVP*, *SOC1*, and *AP1*, whose expression correlates with dormancy transitions [36,40], a comprehensive exploration of MIKC gene involvement in regulating bud dormancy in herbaceous peony is still lacking. 

Given the crucial role of MIKC-type MADS-box genes in regulating bud dormancy and flowering, along with the limited knowledge of their characteristics in herbaceous peony, we aimed to identify and conduct a comprehensive analysis of MIKC genes in *Paeonia lactiflora* Pall. The objective of this study is to achieve the following: (1) identify and characterize the MIKC genes in *Paeonia lactiflora* Pall.; (2) analyze the expression of these MIKC genes during low-temperature-induced BDT and clarify the role of *PlSOC1* in this process. This study offers novel insights into the role of MIKC-type genes in regulating bud dormancy and lays foundation for developing low-chilling requirement herbaceous peony cultivars to address agricultural challenges posed by climate change.

## 2. Results

### 2.1. Identification and Phylogenetic Analysis of MIKC-Type Genes in Herbaceous Peony

A total of 19 MIKC-type transcripts, corresponding to eight genes, were identified from the *Paeonia lactiflora* Pall. transcriptome (Table 1). Detailed information on PlMIKCs, including protein size, molecular weight, isoelectric point (PI), instability index, hydrophilic index (grand average of hydropathy, GRAVY), and subcellular localization is provided in Table 1. Specifically, the lengths of PlMIKC proteins ranged from 127 to 351 amino acids, with molecular weights spanning from 14.30 to 39.55 kDa. The pI values ranged between 5.65 and 10.17. Subcellular localization predictions indicated that all PlMIKCs are localized in the nucleus (Table 1).

**Figure 1:**
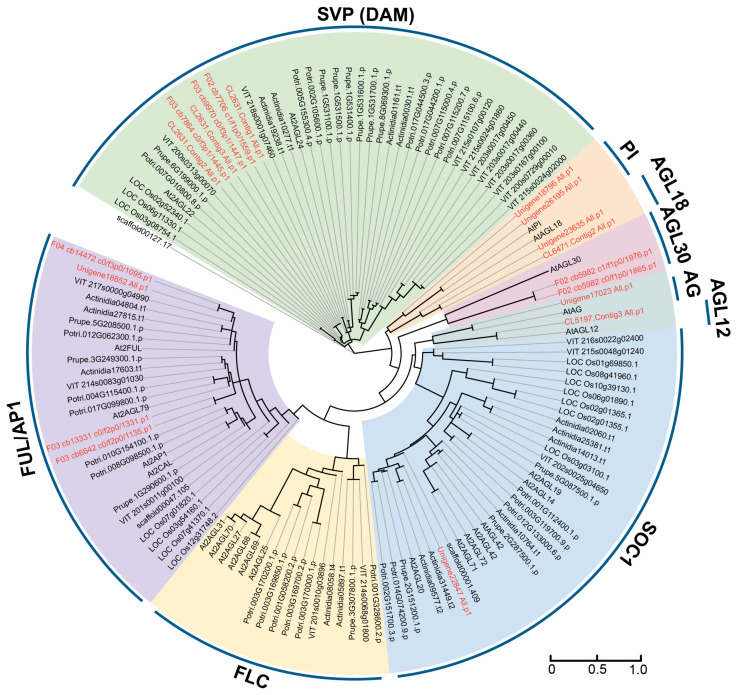
Phylogenetic analysis of PlMIKC proteins and homologs from other plants. The phylogenetic tree was generated by the MEGA v7.0.21 [41] using the maximum likelihood method with 1000 bootstrap replications. *Arabidopsis thaliana* (At), *Vitis vinifera* (VIT), *Populus trichocarpa* (Potri.), *Amborella trichopoda* (scaffold), *Oryza sativa* (LOC_Os), *Actinidia chinensis* (Actinidia), and *Prunus persica* (Prupe.). Different subfamilies are distinguished by different background colors and the PlMIKCC proteins were marked with red colors.

To explore the evolutionary relationships among MIKC-type gene families, a phylogenetic tree was constructed using MIKC proteins from *Arabidopsis thaliana* (At), *Vitis vinifera* (VIT), *Populus trichocarpa* (Potri.), *Amborella trichopoda* (scaffold), *Oryza sativa* (LOC_Os), *Actinidia chinensis* (Actinidia), and *Prunus persica* (Prupe.). The analysis revealed that these proteins were categorized into seven clades, with six classified as MIKC^C^-type, including FUL/AP1, DAM, PI, AGL18, AGL12, AG, and SOC1, and one, AGL30, classified as MIKC*-type (Figure 1). Notably, the FLC subfamily genes were absent in *Paeonia lactiflora* Pall. The DAM (SVP) subfamily had the largest number of members, with six genes, followed by four members in the AP1/FUL-like subfamily, and two genes in the AGL18, PI, and AGL30 subfamilies. The SOC1, AG, and AGL12 subfamilies contained only one gene (Figure 1).

### 2.2. Multiple-Sequence Alignment of PlMIKCs

**Figure 2:**
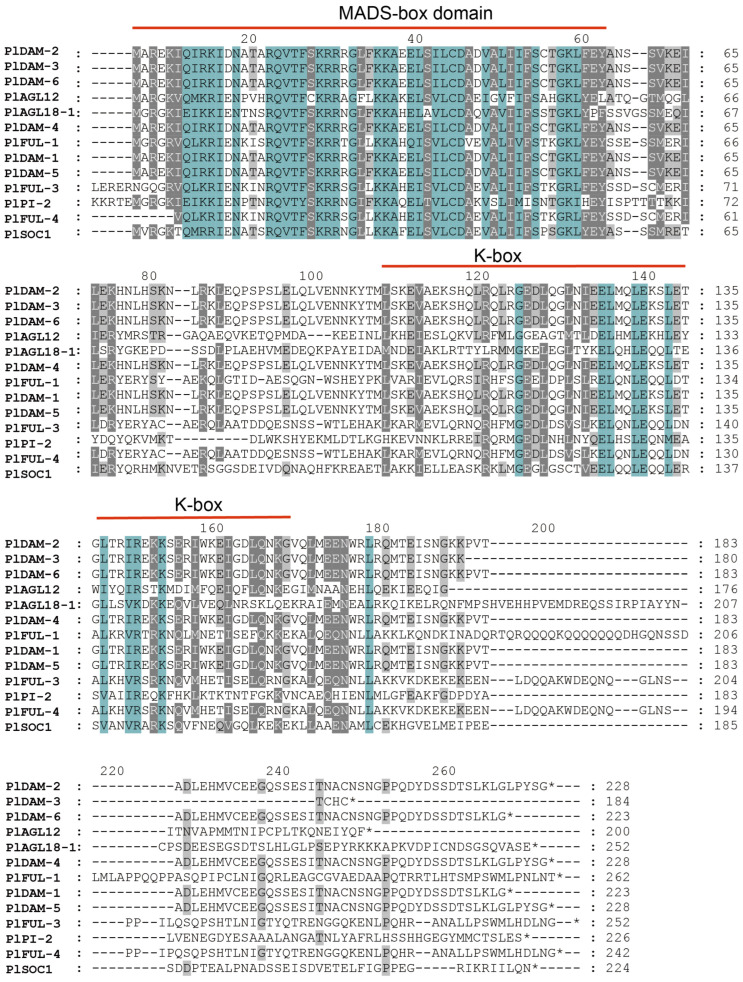
Multiple sequence alignment of key PlMIKC proteins. The MADS and K-domains are indicated with red lines. Background colors denote sequence homology: blue for 100%, dark gray for >75%, and light gray for >50%, respectively.

To further investigate the structures of MIKC-type genes, sequence alignments were conducted on PlMIKC proteins from the AP1/FUL, DAM, SOC1, PI, AGL18, and AGL12 clades. The multiple sequence alignment of these PlMIKCs revealed high similarity among the sequences, which contained the conserved MADS and K-domains. In detail, the MADS domain ranged from approximately 6 to 63 amino acids, while the K-domain spanned from 109 to 144 amino acids (Figure 2).

### 2.3. Protein Structure and Conserved Motif Distribution Analysis

**Figure 3:**
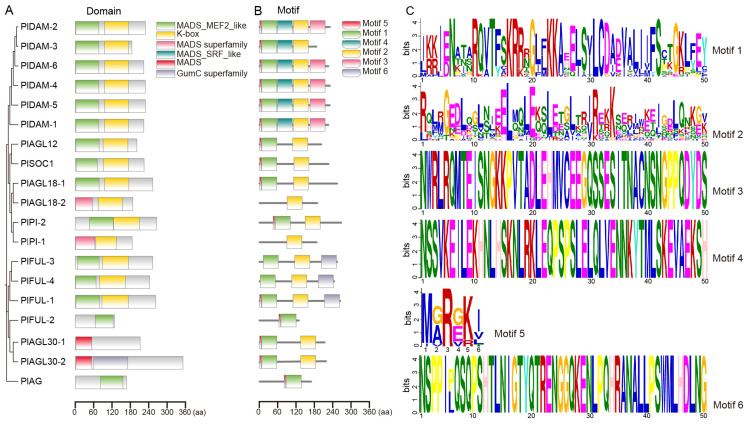
The phylogenetic tree, protein structure, and conserved motifs of the 19 PlMIKC proteins. (**A**) Phylogenetic tree and protein structure. (**B**) Conserved motif. (**C**) Amino acid sequence of the six motifs.

To gain a deeper insight into the structural diversity of PlMIKC genes, protein structure and conserved motifs were compared in relation to their phylogenetic relationships. A phylogenetic tree of the 19 PlMIKC proteins was constructed using the Maximum Likelihood method, revealing four distinct groups: Group 1 consisted of six PlDAMs, Group 2 included PlAGL12, PlAGL18, PlSOC1, and PlPIs, Group 3 contained PlFULs and PlAGL30s, while Group 4 consisted solely of PlAG (Figure 3A). The conserved domain analysis showed that all the 19 PlMIKCs contained the MADS domain, with most also containing the K-domain, except for PlFUL-2, PlAGL30s, and PlAG (Figure 3A). The MEME was employed to detect conserved motifs within the PlMIKC proteins (Figure 3B) and the amino acid sequences of the six conserved motifs are presented in Figure 3C. Motif analysis revealed that motif 1 was the most characteristic MADS-box domain, while motif 2 corresponded to the K-domain. Most PlMIKCs contained both motifs 1 and 2; however, motif 1 was absent in PlAGL18-2 and PlPI-1, while motif 2 was missing in PlFUL-2 and PlAG. In the PlDAM subfamily, members except PlDAM-3 contained motifs 3, 4, and 5. Notably, motif 6 was found only in PlFUL-1, PlFUL-3, and PlFUL-4 among the 19 PlMIKCC proteins, highlighting the structural diversity across subfamilies (Figure 3B,C). These findings suggest that members within the same subfamily share strong similarity in motif composition, indicating that genes within a group may perform similar functions.

### 2.4. Low-Temperature Triggers BDT in Herbaceous Peony

**Figure 4:**
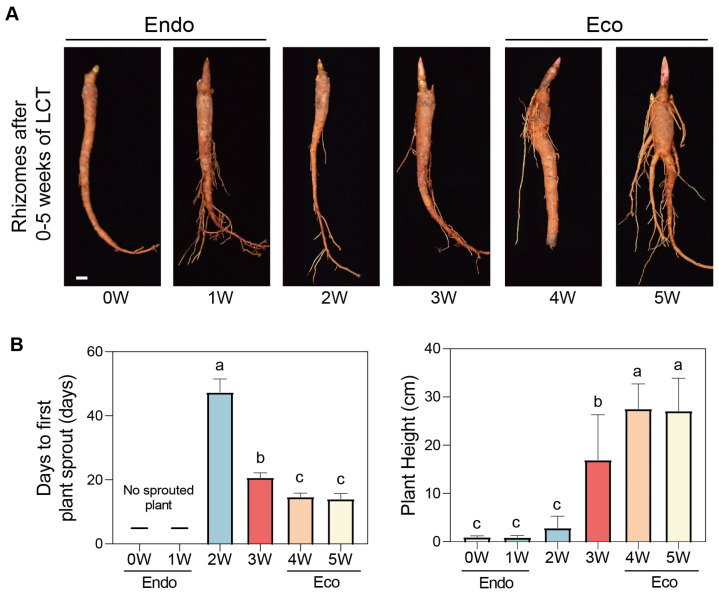
Low temperature induces BDT in herbaceous peony. (**A**) The status of rhizomes after 0–5 weeks of controlled low-temperature treatment. Bar: 1 cm. (**B**) Observations of days to first plant sprouting and plant height of ‘Hang Baishao’ rhizomes which were treated with low temperature exposure for 0–5 weeks and then transferred to greenhouse for 30 days. Endo, endodormancy; Eco, ecodormancy. Data are presented as mean ± SD and Error bars represent SDs. One-way analysis of variance (ANOVA) was used to compare the differences and different letters indicate significant differences (*p* < 0.05).

To explore the involvement of these PlMIKC genes in the dormancy transition of herbaceous peony, we conducted a controlled low-temperature-induced BDT experiment. One-year-old rhizomes of *Paeonia lactiflora* Pall. ‘Hang Baishao’ were placed in a dark freezer at 4 °C. After 0–5 weeks of controlled low temperature exposure, the status of the rhizomes is shown in Figure 4A. The corresponding ability to undergo bud breakage and regrow increased gradually. Specifically, no plants sprouted without chilling treatment or after only 1 week of a short-term low temperature. With prolonged chilling, sprouting occurred more quickly, and plant height increased, but no further significant changes were observed beyond 4 weeks of treatment (Figure 4B). These results indicate that the buds remained in endodormancy during weeks 0 to 1 and shifted to ecodormancy during weeks 4 to 5.

### 2.5. Expression Pattern of the PlMIKC Genes During BDT

**Figure 5:**
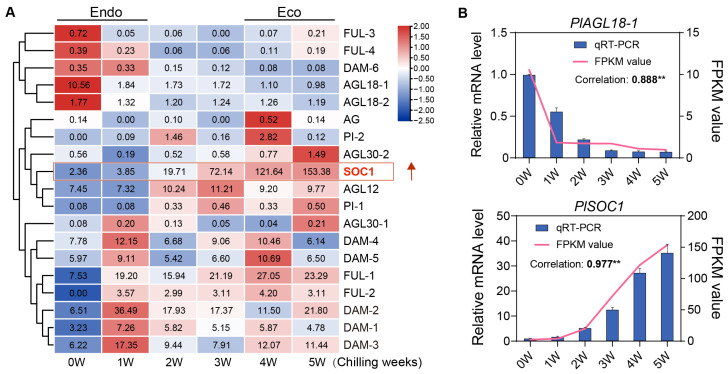
Expression pattern of PlMIKC genes during BDT of herbaceous peony and qRT-PCR analysis of *PlAGL18-1* and *PlSOC1*. (**A**) Expression heatmap of PlMIKC genes during BDT. The numbers in the heatmap represent relative expression FPKM (Fragments Per Kilobase of transcript per Million mapped reads) values. The red arrow and box highlight the significant upregulation of *PlSOC1* during the BDT of herbaceous peony. (**B**) qRT-PCR analysis of *PlAGL18-1* and *PlSOC1*. Endo, endodormancy; Eco, ecodormancy. ** *p* < 0.01.

We next investigated the expression pattern of the 19 PlMIKC genes of ‘Hang Baishao’ during BDT. Cluster analysis revealed three distinct expression trends across the dormancy phases. Several genes, including *PlFUL-3*, *PlFUL-4*, *PlDAM-6*, *PlAGL18-1*, and *PlAGL18-2*, exhibited high expression at week 0 (endodormancy stage), followed by significant downregulation, maintaining consistently low expression levels during the subsequent BDT and ecodormancy phases (Figure 5A). In contrast, genes such as *PlAG*, *PlPI-1*, *PlPI-2*, *PlAGL30-2*, *PlSOC1*, and *PlAGL12* demonstrated low expression during week 0 to week 1, corresponding to the endodormancy phase, but their expression gradually increased, peaking during the late BDT or ecodormancy phases. Another subset of genes, including five *PlDAMs*, two *PlFULs*, and *PlAGL30-1*, showed pronounced upregulation after 1 week of short-term chilling, followed by a decline during the BDT phase. In the ecodormancy phase, their expression either remained low or increased again (Figure 5A). These differential expression patterns underscore the complex regulatory roles of PlMIKC genes during the transition from endodormancy to ecodormancy, reflecting their involvement in the modulation of dormancy phases.

To verify the RNA sequencing results, qRT-PCR was performed on *PlAGL18-1* and *PlSOC1*, which were downregulated and upregulated during BDT, respectively. As shown in Figure 5B, *PlAGL18* was highly expressed during endodormancy but dropped sharply after cold treatment, remaining low in the dormancy transition and ecodormancy stages. In contrast, *PlSOC1* showed a continuous increase under cold exposure. The Pearson correlations between FPKM values and qRT-PCR results were 0.888 and 0.977 (*p* < 0.01), confirming the reliability of the transcriptome data (Figure 5B).

**Figure 6:**
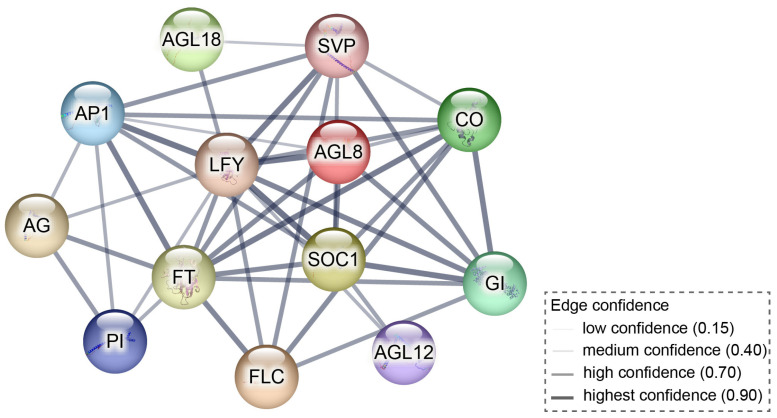
The protein–protein interaction network of *Arabidopsis* MIKC proteins. All interactions are experimentally determined, with line thickness indicating the strength of data support.

To investigate the potential interactions between PlMIKC proteins, we conducted the protein–protein interaction predictions using key *Arabidopsis* MIKC homologous proteins as a reference. In this interaction network, AtSOC1 occupies a central position and exhibits strong interactions with AtFUL (AtAGL8), AtAGL12, AtAP1, and AtSVP, all of which have been reported to interact directly (Figure 6). In this study, *PlSOC1* expression was consistently upregulated under low-temperature treatment, correlating with dormancy transition and positively associated with *PlAGL18* and *PlAGL30* expression, while negatively correlated with the *PlDAMs* and *PlFULs* expression (Figure 4 and Figure 6). These findings suggest that *PlSOC1* may play a regulatory role in herbaceous peony dormancy transition by interacting with *PlFUL*, *PlDAM*, *PlAGL30*, and *PlAGL18*.

### 2.6. PlSOC1 Promotes BDT in Herbaceous Peony

**Figure 7:**
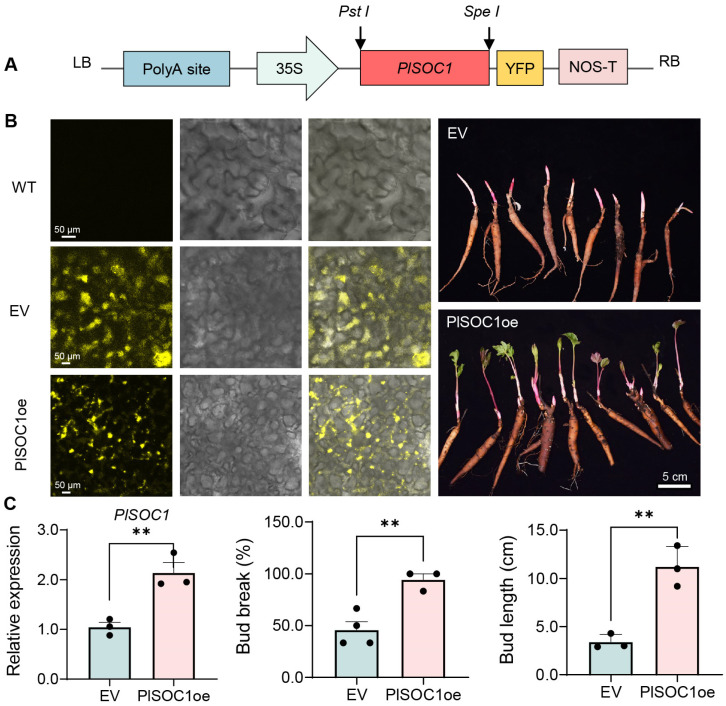
Overexpression of *PlSOC1* promotes dormancy transition and bud break in herbaceous peony. (**A**) YFP-tagged overexpression vector used in this study. (**B**) YFP fluorescence detection and phenotype of transformed plants. (**C**) The expression level of *PlSOC1* and observations of bud break percentage and bud length in *PlSOC1*-overexpressing lines and empty vector-transformed lines. EV, empty vector. Significant differences from the control were determined by Student’s *t*-test (** *p* < 0.01).

The previous study indicates that *PlSOC1* could be essential in regulating the dormancy transition in herbaceous peony (Figure 5 and Figure 6). To further assess its function in dormancy transition in herbaceous peony, we constructed an overexpression vector (Figure 7A) and transiently overexpressed *PlSOC1* in ‘Hang Baishao’ during the early BDT stage (after 3 weeks of low-temperature treatment). YFP fluorescence was detected in *PlSOC1*-overexpressing plants and empty vector-transformed controls, but not in wild-type (WT) plants (Figure 7B). 

Compared to empty vector-transformed plants, *PlSOC1*-overexpressing plants exhibited a significant increase in *PlSOC1* expression levels (Figure 7C). Importantly, the bud break percentage and bud length of *PlSOC1*-overexpressing plants significantly increased from 45.8% to 94.4% and from 3.4 cm to 11.2 cm, respectively (Figure 7B,C). These findings suggest that *PlSOC1* promotes BDT and bud break in herbaceous peony.

## 3. Discussion

### 3.1. MIKC-Type Genes in Peony

With the completion of genome and transcriptome sequencing, MADS-box genes have been extensively identified and analyzed in many significant plant species [42]. The number of MADS-box genes varies significantly across plant species. *Arabidopsis* has been found to contain 107 MADS-box genes, of which 39 are classified as MIKC-type [13]. Similarly, rice (*Oryza sativa*) possesses 75 MADS-box genes, with 38 of them belonging to the MIKC-type [43]. In rose (*Rosa chinensis*), Liu et al. identified 58 non-redundant MIKC^C^ uni-transcripts from transcriptomic data and grouped them into 12 clades [14]. However, the identification and annotation of *MIKC* genes in herbaceous peony remain unexplored. In this study, 19 MIKC-type genes were identified from the herbaceous peony transcriptome and categorized into seven subfamilies based on bioinformatics analysis: FUL/AP1 (4), SVP (6), AGL18 (2), PI (2), AGL30 (2), AG (1), AGL12 (1), and SOC1 (1). Interestingly, the FLC subfamily was absent from the *Paeonia lactiflora* Pall. transcriptome, a finding consistent with reports from other plant species, such as wintersweet (*Chimonanthus praecox*) [44] and rose [14]. Such losses of MIKC genes, particularly within the TM8-like and FLC-like clades, are hypothesized to reflect adaptive evolutionary events [45]. 

Tree peony (*Paeonia* sect. *Moutan* DC.) is a perennial deciduous shrub and a sibling species of herbaceous peony belonging to the same genus *Paeonia*. Since the publication of the tree peony (*Paeonia ostii*) genome [46], the genome-wide characterization of the MADS-box gene family in *Paeonia ostii* has been conducted. Yang et al. identified 110 MADS-box genes in *Paeonia ostii*, among which 40 were MIKC genes. MIKC genes were further classified into 12 evolutionary clades: AP1/FUL, AP3/PI, AG, SEP, SOC1, SVP, AGL6, AGL12, AGL15, AGL17, FLC, and BS. Notably, no PoMIKC genes were found in the FLC and AGL17 clades, similar to the findings in this study [47]. In both herbaceous and tree peony, the MIKC gene family is an important factor in regulating flower development. In *Paeonia lactiflora* Pall., *PlAP3-1*, *PlAP3-2*, and *PlPI* mainly govern stamen and petal formation, while *PlSEP3* controls sepal and petal identity. The expression analysis of key MIKC genes in *Paeonia ostii* floral organs showed that MIKC genes play a crucial role in floral development [47]. In tree peony (*Paeonia suffruticosa*), *PsAGL6*, *PsAG*, and *PsSEP* likely regulate sepals and carpels, while *PsPI* probably controls stamen and pistil petalization [48,49,50].

### 3.2. Functional Analysis of PlMIKC Genes Based on Gene Expression Pattern

The MADS-box gene family, particularly with regard to the MIKC-type genes, has been widely recognized as key regulators of dormancy transitions [11,44]. The expression trends of the 19 PlMIKCs genes during the BDT of ‘Hang Baishao’ in this study provide new insights into their roles in dormancy regulation. Importantly, *PlDAMs* were highly expressed during endodormancy but were downregulated during the BDT phase. This suggests their role in dormancy maintenance, similar to the function of *DAMs* in species such as pear [20] and apricot [21]. Meanwhile, *FUL* (*AGL8*), a member of MIKC genes acting downstream of *FT*, has been shown to interact with *SOC1* to promote flowering and modulate meristem determinacy in *Arabidopsis* [51]. Although the expression trends of *PlFUL*-1 and *PlFUL-2* during endodormancy were similar to that of *PlDAM*, with both showing significant upregulation, *PlFUL* exhibited a gradual increase throughout the BDT and ecodormancy phases. This suggests that *PlFUL* might be involved in the dormancy transition of herbaceous peony in response to prolonged low temperature exposure. Additionally, the transient upregulation of several *PlDAM* and *PlFUL* genes in response to short-term low-temperature highlights the responsiveness of MIKC-type genes to environmental cues, as observed in other perennial species like peach [18] and pear [20].

In contrast to *PlDAMs*, *PlSOC1*, *PlAGL12*, and *PlAGL30* exhibit low expression during endodormancy but show increased expression during BDT, consistent with dormancy transition phases. In *Arabidopsis*, *AGL18* is pivotal for root development and flowering transition [31], while *AGL30* plays a key role in regulating pollen development [52]. However, their specific contributions to dormancy regulation remain largely unexplored. Notably, in this study, *AGL18* and *AGL30* displayed expression patterns similar to *SOC1* and were positively correlated with BDT, suggesting a potential positive regulatory role in BDT. These findings highlight the diverse and critical functions of MIKC genes in dormancy regulation, offering valuable insights into their potential roles in orchestrating developmental transitions during dormancy phases.

### 3.3. SOC1: Traditionally Known for Promoting Flowering, but Also Facilitates Dormancy Transition

SOC1 is known as a central regulator in *Arabidopsis* floral transition and flowering time by integrating multiple signaling pathways [32]. The overexpression of the tree peony *PsSOC1* gene in *Arabidopsis* promoted early flowering, accompanied by the upregulation of *LEAFY* (*LFY*) [53]. The *Bambusa oldhamii SOC1-like* gene *BoMADS50* was highly expressed during the floral primordium formation stage, and its overexpression induced early flowering in transgenic rice [54]. Nevertheless, the role of *SOC1* in the bud dormancy transition of perennials remains largely unknown. However, seasonal expression studies have detected *SOC1* activity during winter dormancy in several species, such as tree peony [53] and Japanese apricot [55]. Voogd et al. [23] investigated the phenotype of kiwifruit lines overexpressing *AcSOC1i*, *AcSOC1e*, and *AcSOC1f*, revealing that only *AcSOC1i* significantly promoted earlier bud break compared to control plants [23]. Similarly, hybrid poplars in the ecodormancy stage overexpressing *MADS12* (a *SOC1-like* gene) show early bud break, highlighting the involvement of *SOC1* in bud reactivation [56]. A recent study in tree peony confirmed that PsSOC1 interacts with the DELLA protein RGA-LIKE 1 (RGL1), a negative regulator of the GA pathway, to promote bud dormancy release [22]. In addition, *SOC1* is believed to be associated with chilling requirements. In apricot, specific allele combinations of *SOC1*, such as 262/262 or 262/278, have been linked to high chilling requirement trait, while the 215/278 combination is associated with low-CR trait [57]. Our earlier research on herbaceous peony observed the consistent upregulation of *PlSOC1* until the ecodormancy phase during winter [36,40]. In this study, *PlSOC1* was the most highly expressed gene among the 19 MIKC genes, with its expression positively correlated with dormancy transition. Transient overexpression of *PlSOC1* in ‘Hang Baishao’ significantly promoted bud break. Therefore, an increasing number of studies have shown that *SOC1* is not only an integrator of flowering but also a key factor in promoting dormancy transition. 

## 4. Materials and Methods

### 4.1. Plant Materials

**Figure 8:**
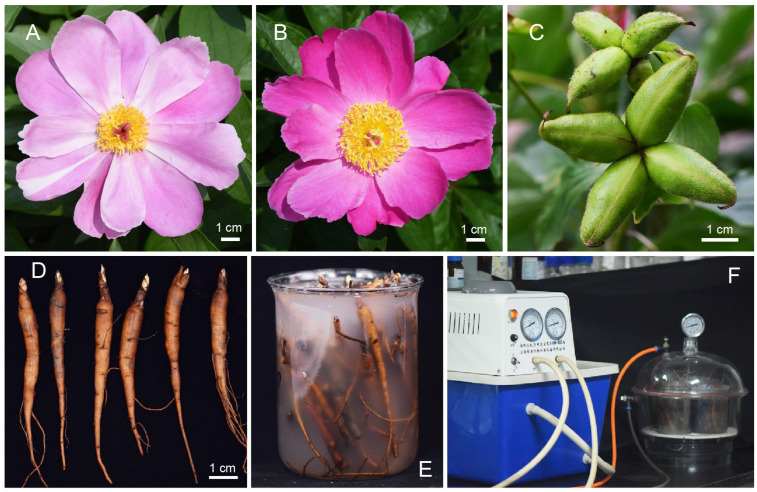
Flowers, seed pod, and one-year-old rhizomes of plant material *Paeonia lactiflora* Pall. ‘Hang Baishao’. The flowers of ‘Hang Baishao’ display a range of colors, with pink (**A**) and red-purple (**B**) being the most prevalent. One-year-old rhizomes (**C**) offer the advantages of being easier to obtain, smaller in size (**D**), and more suitable for *Agrobacterium* immersion (**E**) and infection (**F**).

The *Paeonia lactiflora* Pall. cultivar ‘Hang Baishao’ was selected as plant material based on our previous research [36,37]. ‘Hang Baishao’ is a native cultivar with a low chilling requirement trait and can grow successfully in low-latitude regions (Figure 8) [38,58].

The flowers of ‘Hang Baishao’ display a range of colors, with pink (Figure 8A) and red-purple (Figure 8B) being the most prevalent. In addition, it produces large and abundant seed pods (Figure 8C), making it a highly valuable oilseed crop. As a geophyte, the aboveground parts of ‘Hang Baishao’ senesce in September, entering a dormant phase as underground buds during the winter. In the following spring, ‘Hang Baishao’ resumes growth in March and blooms in mid-April [36,38]. Moreover, we have conducted extensive agronomic observations on ‘Hang Baishao’ over the years. For more details on its chilling requirement, adaptability, ornamental features and growth habits, please refer to Wang et al. [37,38]. 

In this study, dormant one-year-old ‘Hang Baishao’ rhizomes are used for cold treatment and gene function verification. ‘Hang Baishao’ rhizomes were obtained from the Perennial Flower Resources Garden at Zhejiang University in Hangzhou, China. Compared to multi-year-old herbaceous peony, one-year-old rhizomes offer advantages of being easier to obtain, smaller in size (Figure 8D), and more suitable for *Agrobacterium* infection (Figure 8E,F) [59].

### 4.2. Low-Temperature Treatment and Transcriptome Analysis

Dormant one-year-old ‘Hang Baishao’ rhizomes were subjected to a controlled low temperature (4 °C) for 0 to 5 weeks (0–5 W) to obtain rhizome buds at the endodormancy, dormancy transition, and ecodormancy stages, respectively. The buds from different dormancy stages were collected, immediately frozen in liquid nitrogen, and stored at −80 °C until subsequent transcriptome sequencing. Additionally, a group of rhizomes, exposed to 0–5 weeks of low temperature, was transferred to a greenhouse (25 °C, 16/8 h light/dark) to observe bud break and growth performances, as described by Wang et al. [36].

Total RNA was extracted from the sampled buds using the RNAprep Pure Plant Kit (Tiangen, Beijing, China). The RNA concentration and quality were measured using a Nanodrop 2100 spectrophotometer (Thermo Fisher Scientific, Waltham, MA, USA), and its integrity was assessed by 1.0% agarose gel electrophoresis. Subsequently, cDNA libraries were constructed using a SMARTer PCR cDNA Synthesis Kit (Clontech, Mountain View, CA, USA). The final library, prepared at a volume of 20 µL with a concentration of 5 nM, was sequenced on the BGIseq500 platform (BGI, Beijing Genomic Institute, Shenzhen, China) with three biological replicates per sample. Sequencing data were filtered using SOAPnuke (v1.5.2) to yield clean reads in FASTQ format. Since the genome of herbaceous peony has not yet been published, clean reads were de novo assembled into transcripts using Trinity v2.5.1. Gene expression was normalized into FPKM using RSEM (v.1.3.0). 

### 4.3. Identification and Phylogenetic Analysis of MIKC Genes

To identify MIKC genes, a separate phylogenetic analysis was conducted. Specifically, a hidden Markov model (HMM) search was performed on the herbaceous peony transcriptome using the HMM profile (PF00319) of the SRF-TF domain from the Pfam database (http://pfam.xfam.org/, accessed on 2 January 2025). MADS-box (PF00319) HMM searches were conducted with HMMER3.0 [60] with default parameters (http://hmmer.org/, accessed on 2 January 2025). Additionally, protein sequences were also compared to representative MIKC genes from *Arabidopsis* using BLASTP with an E-value cutoff of 10^−5^ to detect potential MIKC genes absent from the predicted protein set. Low-quality sequences lacking both start and stop codons were excluded to improve accuracy. The identified MIKC genes were further verified through the NCBI Conserved Domain Database (https://www.ncbi.nlm.nih.gov/cdd, accessed on 2 January 2025).

A phylogenetic analysis of MIKC proteins was carried out using the predicted proteomes of *Paeonia lactiflora* Pall. ‘Hang Baishao’ along with representative plant species: *Arabidopsis thaliana*, *Vitis vinifera*, *Populus trichocarpa*, *Amborella trichopoda*, *Oryza sativa*, *Actinidia chinensis*, and *Prunus persica*. Among these species, *Arabidopsis thaliana* and *Oryza sativa* are representative dicot and monocot species, respectively. *Populus trichocarpa* serves as a model for bud dormancy in woody plants, while *Amborella trichopoda* represents the most basal angiosperm lineage. *Actinidia chinensis*, *Prunus persica*, and *Vitis vinifera* are key fruit tree species, with well-established research on their role in MIKC *genes* during bud dormancy [16,23,61]. Protein sequences of *Arabidopsis thaliana* MIKC family members were downloaded from The Arabidopsis Information Resource (TAIR, http://www.Arabidopsis.org/, accessed on 3 January 2025). Sequences of *Actinidia chinensis* were obtained from the Kiwifruit Genome Database (http://kiwifruitgenome.org/, accessed on 3 January 2025). Supplementary genome and protein data were obtained from NCBI and Phytozome (https://phytozome.jgi.doe.gov/, accessed on 3 January 2025). MIKC protein sequences were aligned using MAFFT v7.467 [62] with default settings. The phylogenetic analysis of the alignment was performed with FastTree v2.1.12 [63]. The phylogenetic tree based on sequence similarity was generated and visualized using MEGA v7.0.21 and the sequence alignment of PlMIKCs was visualized using GeneDoc v2.6 [64].

### 4.4. Physicochemical Characteristics and Subcellular Localization Prediction

The physicochemical characteristics of PlMIKC proteins were assessed via ExPASY ProtParam (https://web.expasy.org/protparam/, accessed on 7 January 2025). The analysis included parameters such as molecular weight, amino acid composition, instability index, PI, and GRAVY score. Subcellular localization predictions were conducted using Plant-mPLoc (http://www.csbio.sjtu.edu.cn/bioinf/plant-multi/, accessed on 7 January 2025) [65].

### 4.5. Protein Structure and Conserved Motif Analysis

Domain information for the PlMIKCs was identified using NCBI Batch-CD-search (https://www.ncbi.nlm.nih.gov/Structure/bwrpsb/bwrpsb.cgi, accessed on 7 January 2025) [66]. Conserved motifs within the PlMIKC proteins were examined through MEME Suite (V5.5.7, http://meme-suite.org, accessed on 7 January 2025) [67]. Additionally, TBtools v2.152 was employed to visualize the phylogenetic tree, domains, and conserved motifs of the 19 PlMIKC proteins [68].

### 4.6. Expression Profiles of PlMIKC Genes During Dormancy Transition

The expression levels of PlMIKC genes during dormancy transitions were visualized in TBtools v2.152 using log2-transformed FPKM values from the transcriptome obtained in 4.1. The protein–protein interaction network was analyzed using the STRING database (https://cn.string-db.org/, accessed on 7 January 2025) [69].

### 4.7. Transient Overexpression of PlSOC1 in Herbaceous Peony

The PlSOC1 coding sequence was cloned into the pHB-YFP vector to create the *p35S::PlSOC1* construct, which was subsequently transferred into *Agrobacterium* strain GV3101. The *Agrobacterium* cells were cultured until the bacterial suspension reached an OD_600_ of 1.2, then centrifuged at 4000 rpm for 10 min at 25 °C. The pellet was resuspended in infection buffer (OD = 1.0) containing 10 mM MgCl_2_, 10 mM 2-(N-morpholino) ethanesulfonic acid (MES), and 20 µM acetosyringone (AS), and subsequently incubated at 28 °C in the dark for 3 h. One-year-old *Paeonia lactiflora* Pall. rhizomes, chilled for 3 weeks, were immersed in the incubated *Agrobacterium* suspension and subjected to a negative pressure vacuum (−0.9 kg/cm^2^) for 10 min, followed by slow deflation for 20 min. After infection, the rhizomes were rinsed with clean water for 5 min, dried with absorbent paper, and initially kept in darkness for 3 days before being transferred to a growth chamber for incubation (22 °C, 16/8 h light/dark cycle, and light intensity of 2000 lux). After 15 days of cultivation, transformed rhizome buds were collected to observe phenotypes and detect YFP fluorescence and *PlSOC1* expression. Plants showing both YFP fluorescence and a significant increase (*p* < 0.05) in *PlSOC1* expression were confirmed as positively transformed. The primers used are listed in Table 2.

### 4.8. Quantitative Real-Time PCR (qRT–PCR) Analysis

One microgram of the extracted total RNA from Section 4.2 was converted to cDNA using the PrimeScript RT Reagent Kit with gDNA Eraser (Takara, Shiga, Japan). *Alpha-tubulin* (*TUBA*) was used as a reference gene [36] and the primers are listed in Table 3. qRT–PCR was conducted with SYBR Premix Ex Taq (Takara, Shiga, Japan) on a CFX Connect TM Real-Time PCR Detection system (Bio-Rad, Hercules, CA, USA). Every reaction contained 4 µL of cDNA, 5 µL of TB Green, 0.5 µL of forward primer, and 0.5 µL of reverse primer. The qRT–PCR cycle parameters were as follows: 2 min at 95 °C; 39 cycles of 5 s at 95 °C and 30 s at 55 °C; and a melting curve program of 5 s at 95 °C, 5 s at 65 °C, and 5 s at 95 °C. The PCR procedure used was completed according to the manufacturer’s instructions. The 2^−△△CT^ method was used to calculate the gene expression level [70]. 

## 5. Conclusions

In this study, we identified and conducted a phylogenetic analysis of MIKC-type genes in herbaceous peony. A total of 19 PlMIKC genes were identified and classified into seven subfamilies, with the FLC subfamily notably absent in the *Paeonia lactiflora* Pall. transcriptome. We analyzed the domain information, conserved motif distribution, and expression patterns of these PlMIKC genes during different dormancy stages in herbaceous peony. The findings highlighted the diverse and crucial roles of *PlDAM*, *PlFUL*, *PlSOC1*, and *PlAGL18* in dormancy transition. Furthermore, the overexpression of *PlSOC1* in herbaceous peony confirmed its role in promoting dormancy transition and bud break. This study fills the gap in the comprehensive analysis of MIKC-type genes in herbaceous peony and enhances the understanding of their role in dormancy regulation. Additionally, it provides a foundation for breeding low-chilling-requirement peony germplasm for cultivation in warming climates.

## Figures and Tables

**Table 1:** Detailed information on the 19 MIKC-type genes identified from herbaceous peony.

Gene Name	Gene ID	Amino Acid	Molecular Weight	PI	Instability Index	GRAVY	Subcellular Location
PlDAM-2	CL2631.Contig1_All	228	26,050.66	6.92	59.02	−0.734	Nucleus
PlDAM-3	CL2631.Contig2_All	184	21,467.82	9.26	59.32	−0.723	Nucleus
PlDAM-6	CL2631.Contig3_All	223	25,533.08	6.92	59.25	−0.749	Nucleus
PlAGL12	CL5197.Contig3_All	200	23,194.02	7.02	47.74	−0.408	Nucleus
PlAGL18	CL6471.Contig2_All	252	28,738.64	6.04	54.01	−0.762	Nucleus
PlAGL30-1	F02_cb5982_c0/f1p0/1865	211	24,086.83	9.82	38.24	−0.559	Nucleus
PlAGL30-2	F02_cb5982_c1/f1p0/1976	351	39,547.65	6.12	49.89	−0.663	Nucleus
PlDAM-4	F02_cb7706_c1/f1p0/1559	228	26,050.66	6.92	59.02	−0.734	Nucleus
PlFUL-1	F03_cb13331_c0/f2p0/1331	262	30,262.34	9.55	58.59	−0.929	Nucleus
PlFUL-2	F03_cb6642_c0/f2p0/1135	127	14,302.88	10.01	40.17	0.035	Nucleus
PlDAM-1	F03_cb7864_c0/f3p1/1445	223	25,533.08	6.92	59.25	−0.749	Nucleus
PlDAM-5	F03_cb9970_c0/f3p1/1447	228	26,050.66	6.92	59.02	−0.734	Nucleus
PlFUL-3	F04_cb14472_c0/f3p0/1095	252	29,265.89	8.68	56.03	−1.028	Nucleus
PlAG	Unigene17023_All	168	19,064.71	10.17	47.30	−0.702	Nucleus
PlPI-2	Unigene18796_All	265	30,655.26	9.35	37.83	−0.609	Nucleus
PlFUL-4	Unigene18852_All	242	28,053.55	8.38	56.58	−0.992	Nucleus
PlSOC1	Unigene22847_All	224	25,382.85	5.65	54.89	−0.637	Nucleus
PlAGL18-2	Unigene23635_All	188	21,515.76	5.86	58.56	−0.422	Nucleus
PlPI-1	Unigene26195_All	185	21,385.22	6.10	55.92	−0.764	Nucleus

Note: PI, isoelectric point; GRAVY, grand average of hydropathy.

**Table 2:** Primer sequences used for generating *p35S::PlSOC1* construct in this study.

Primer Names	Primer Sequences (5′-3′)
p35S::PlSOC1_F	CTTGGATCCTCGAGCTGCAGGAAGCTCAATCAGTTTCATCCCAA
p35S::PlSOC1_R	CCCTTGCTCACCATACTAGTGTTCTGTAATATAATGCGTTTAATTCTGC

**Table 3:** Primer sequences used for qRT–PCR in this study.

Primer Names	Primer Sequences (5′-3′)
PlATUBA_F	GACGTGGGCTCGATCTGAAT
PlATUBA_R	ATCCCTTGCCTGAGCATCAC
PlSOC1_F	ATGCGCGAGACAATTGAACG
PlSOC1_R	TCAACGGTGCATGATCCCAA
PlAGL18-1_F	CGCTGCGCAAGCAGATTAAG
PlAGL18-1_R	TAAGGCTCAGAAGGCAACCC

## Data Availability

Data are contained within the article; additional inquiries can be addressed directly to the corresponding authors.
